# The influence of SARS-CoV-2 spike protein exposure on retinal development in the human retinal organoids

**DOI:** 10.1186/s13578-025-01383-0

**Published:** 2025-04-11

**Authors:** Jing Gong, Lingling Ge, Yuxiao Zeng, Cao Yang, Yushan Luo, Jiahui Kang, Ting Zou, Haiwei Xu

**Affiliations:** 1 Southwest Hospital/Southwest Eye Hospital, Third Military Medical University (Army Medical University), Chongqing, 400038 China; 2 Key Lab of Visual Damage and Regeneration & Restoration of Chongqing, Chongqing, 400038 China; 3 Present Address: Centre for Lipid Research & Key Laboratory of Molecular Biology for Infectious Diseases (Ministry of Education), the Second Affiliated Hospital, Chongqing Medical University, Chongqing, 400016, China; 4 Present Address: Department of Ophthalmology, The Second Affiliated Hospital of Chongqing Medical University, Chongqing, 400010 China

**Keywords:** SARS-CoV-2, Retinal development, Spike protein, Retinal organoid

## Abstract

**Background:**

Pregnant women are considered a high-risk population for severe acute respiratory syndrome coronavirus 2 (SARS-CoV-2) infection, as the virus can infect the placenta and embryos. Recently, SARS-CoV-2 has been widely reported to cause retinal pathological changes and to infect the embryonic retina. The infection of host cells by SARS-CoV-2 is primarily mediated through spike (S) protein, which also plays a crucial role in the pathogenesis of SARS-CoV-2. However, it remains poorly understood how the S protein of SARS-CoV-2 affects retinal development, and the underlying mechanism has not yet been clarified.

**Methods:**

We used human embryonic stem cell-derived retinal organoids (hEROs) as a model to study the effect of S protein exposure at different stages of retinal development. hEROs were treated with 2 μg/mL of S protein on days 90 and 280. Immunofluorescence staining, RNA sequencing, and RT-PCR were performed to assess the influence of S protein exposure on retinal development at both early and late stages.

**Results:**

The results showed that ACE2 and TMPRSS2, the receptors facilitating SARS-CoV-2 entry into host cells, were expressed in hEROs. Exposure to the S protein induced an inflammatory response in both the early and late stages of retinal development in the hEROs. Additionally, RNA sequencing indicated that early exposure of the S protein to hEROs affected nuclear components and lipid metabolism, while late-stages exposure resulted in changes to cell membrane components and the extracellular matrix.

**Conclusion:**

This work highlights the differential effects of SARS-CoV-2 S protein exposure on retinal development at both early and late stages, providing insights into the cellular and molecular mechanisms underlying SARS-CoV-2-induced developmental impairments in the human retina.

**Supplementary Information:**

The online version contains supplementary material available at 10.1186/s13578-025-01383-0.

## Background

The coronavirus disease 2019 (COVID-19) pandemic is the one of the most significant public health crises, caused by severe acute respiratory syndrome coronavirus 2 (SARS-CoV-2). In addition to the respiratory system, other systems have also been reported to be affected by SARS-CoV-2 [1–3]. Notably, pregnant women are a high-risk group for SARS-CoV-2 infection, as the placenta and embryo have been widely observed to be infected by the virus [4–6]. Recently, transient neurological complications have been identified in neonates born to mothers infected with SARS-CoV-2, suggesting that the fetal central nervous system (CNS) may be susceptible to the virus [7]. Retina is a part of CNS and plays a critical role in maintaining visual function [8]. It has been reported that SARS-CoV-2 mRNA was detected in the human retina [9]. In addition, retinal complications were observed in patients with COVID-19 [10, 11]. Recent work revealed that infection with SARS-CoV-2 in animal models resulted in retinal inflammation [12]. Unfortunately, little is known about the effects of SARS-CoV-2 infection on the fetal retina at different developmental stages.

Coronavirus infection of  host cells is mainly mediated by the spike (S) protein, a membranous glycoprotein that is essential for viral entry [13]. The S protein interacts with the angiotensin converting enzyme 2 (ACE2); subsequently, the host transmembrane serine protease 2 (TMPRSS2) primes the S protein for cell entry and allows the fusion of the viral membrane with the host cellular membrane [14, 15]. It has been reported that the S protein can upregulate ACE2 expression by enhancing the effects of interferon in bronchial epithelium, potentially facilitating viral entry into host cells [16]. The binding of the SARS-CoV-2 S protein selectively accelerates the substrate-specific catalytic activity of ACE2, which may contribute to cardiovascular complications [17]. In addition, the S protein has been shown to disrupt the vascular and immune regulatory functions of the blood–brain barrier, affect brain homeostasis, and exert neurotoxic effects on the CNS [18–20]. In the eye, ACE2 and TMPRSS2 have been shown to be abundantly expressed on the ocular surface including the conjunctival, corneal, and limbal epithelium [21–23], as well as in posterior segments such as the retina [24, 25]. Due to ethical constraints, the expression patterns of ACE2 and TMPRSS2 receptors, as well as the effects and underlying mechanisms of the S protein in developing human retinal tissues, remain unexplored.

Human pluripotent stem cell (hPSC)-derived organoids are characterized by their ability to mimic the cell-type composition, structure and function, which is extremely attractive for investigation of the basic developmental dynamics, disease models and personalized therapeutic approaches [26], offering a way to overcome the species differences in animal models and the ethical limitations of researching human tissues. Recently, lung organoids have been used as a model to study the underlying mechanisms of SARS-CoV-2 infection and to develop new therapeutic drugs [27–29]. In addition, exposing kidney organoids to SARS-CoV-2 results in the activation of profibrotic signaling pathways, which is similar to kidney fibrosis observed in SARS-CoV-2 patients [30]. To study the influence of SARS-CoV-2 on the CNS, human brain organoids have been used to examine SARS-CoV-2 neurotropism. It was found that SARS-CoV-2 infected choroid plexus cells, disrupting the blood-cerebrospinal fluid barrier in brain organoids [31]. Retinal organoids have also been utilized to investigate retinal development and diseases, including preliminary research on COVID-19. It was demonstrated that ACE2 and TMPRSS2 were expressed in hPSC-derived retinal organoids, suggesting the potential of retinal organoids as a model for SARS-CoV-2 infection [32]. Subsequently, Menuchin-Lasowski et al.'s study  revealed that SARS-CoV-2 infected and replicated in photoreceptors and retinal ganglion cells through the ACE2 pathway, leading to retinal inflammation in the retinal organoids [33]. However, the mechanism by which the SARS-CoV-2 S protein affects developing retinal tissue at different developmental stages remains to be elucidated.

Here, we used human embryonic stem cell-derived retinal organoids (hEROs) as an in vitro model to detect the influence of the S protein and the expression of ACE2 and TMPRSS2 at different stages of retinal development. It was showed that short-term exposure to the S protein at different stages of retinal development did not result in significant differences in ACE2 and TMPRSS2 expression levels, nor in the expression of markers for retinal photoreceptor cells, amacrine cells, or ganglion cells. RNA sequencing data revealed that inflammatory gene expression was altered after S protein treatment at both the early and late stages of retinal organoid development, with the early stage showing activation of lipid metabolism and the late stage primarily affecting the extracellular matrix (ECM). These findings provide new insights into elucidating the influence and mechanisms of SARS-CoV-2 infection on retinal development.

## Methods and materials

### hESC culture and the generation of hEROs

The hESCs (H9 line) [34] were provided by the Stem Cell Bank, Chinese Academy of Sciences (Serial: SCSP-307; https://www.cellbank.org.cn/search-detail.php?id=636). hESCs were cultured on Vitronectin (Gibco)-coat 6-well plates using Essentinal 8^™^ culture medium (Gibco) without feeders. The generation of hEROs followed our previous studies [35, 36]. Briefly, hESCs were dissociated into a single-cell suspension using TryPLE™ Express (Gibco) and quickly reaggregated in 96-well low-cell-adhesion V-bottom plates (MS-9096VZ) in retinal differentiation medium supplemented with 20 μM Y-27632 (Sigma-Aldrich) for 6 days. The retinal differentiation medium contained 45% F12-Glutamax (Gibco), 45% IMDM (Gibco), 450 μM monothioglycerol (Sigma-Aldrich), 1% chemically defined lipid concentrate (Gibco), and 10% knockout serum replacement (KSR, Gibco). On day 6, fresh differentiation medium supplemented with 1.5 nM recombinant human bone morphogenetic protein 4 (BMP4, Peprotech) was used to promote the induction of hEROs. Subsequently, half of the medium was exchanged every three days. After 18 days of induction, hEROs were transferred to ultra-low attachment 6-well plates (Corning) in long-term culture medium [(Dulbecco’s modified Eagle’s medium (DMEM)/F12-Glutamax (Gibco), 1% N2 supplement (Gibco), 10% fetal bovine serum (FBS, Gibco), 0.5 μM RA (Sigma), 0.1 mM taurine (Sigma), and 100 U/ml penicillin and 100 μg/ml streptomycin (Gibco))] containing 3 μM GSK3 inhibitor (Wnt agonist) and 5 μM FGFR inhibitor for 6 days. Finally, the induction-reversal culture medium was replaced with fresh long-term culture medium to obtain mature retinal organoids.

### Spike protein exposure

The S protein (R&D Systems, 10549-CV-100) was applied in present study according to the manufacturer’s instructions. Similar to the S protein concentrations observed in the plasma of some COVID-19 patients (2.5-17.5 ug/mL) [37] ,1, 2, 5, and 10 μg/mL of S protein were supplemented into the long-term culture medium to treat the 90-day hEROs for 48h to determine the suitable exposure concentration.Then, 2 μg/mL of S protein was used to treat the different hEROs at different time points (90 days and 280 days) for 48 h.

### Immunofluorescence staining

Immunostaining of hEROs was performed as described in our previous study [38]. hEROs were randomly harvested and fixed with 4% paraformaldehyde for 30 min at 4 ℃ and then transferred to 30% sucrose for dehydration at 4 ℃. Specimens were embedded in optimal cutting temperature (OCT) compound the following day, and frozen at – 80 ℃ freezer. Using a Leica CM1900UV cryostat (Germany), 12-μm-thick frozen sections were made. For immunofluorescence staining, the OCT compound was removed with PBS, and cryosections were permeabilized in 0.3% (v/v) Triton X-100 at room temperature for 15 min, then, blocking with 1% (w/v) bovine serum albumin, 10% (v/v) goat serum, and 0.1% (v/v) Triton X-100 for 60 min at room temperature. The following primary antibodies, including goat anti-ACE2 (AF933, R&D Systems, 1:200), rabbit anti-TMPRSS2 (ab109131, Abcam, 1:400), rabbit anti-Ki67 (ab15580, Abcam,1:200), rabbit anti-Cleaved Caspase-3 (9661, Cell Signaling Technology, 1:200), mouse anti-CHX10 (sc-374151, Santa Cruz, 1:50), rabbit anti-HuC/D (ab184267, Abcam,1:400), rabbit anti-Recoverin (AB5585, Millipore, 1:500), rabbit anti-Vimentin (ab92547, Abcam, 1:500), and rabbit anti-AP2α (3215, Cell Signaling Technology, 1:200) were diluted in blocking solution. Subsequently, cryosections were incubated with primary antibodies at 4 ℃ overnight. On the following day, the cryosections were watched with 1 × PBS for three times and then incubated with appropriate secondary antibodies (donkey anti-goat IgG Alexa-Fluor-568, ab175474, Abcam, 1:500; goat anti-rabbit IgG Alexa-Fluor-568, A11011, Life Technologies, 1:500; and goat anti-mouse IgG Alexa-Fluor-488, A11001, Life technologies, 1:400) at 37 ℃ for 1 h. After washing the cryosections three times with 1 × PBS, DAPI (C1006, Beyotime) dye was applied for nuclear counterstaining at room temperature. Finally, the cryosections were viewed and photographed using a Zeiss, LSM880 confocal microscope. Images were analyzed and processed using the Image J (64-bit) software. The ratio of positive cells was analyzed by positive cells/area (μm^2^).

### TUNEL assays

Apoptosis of cells within hEROs was assessed by TUNEL assay (C1089, Beyotime) after 48 h of S protein treatment at 90 and 280 days. The TUNEL-positive cells were stained following the manufacturer’s protocols. After the TUNEL reaction solution was incubated at 37 °C for 60 min, DAPI was added for 10 min, followed by washing with PBS three times. The sections were visualized using confocal laser scanning microscopy (Zeiss, LSM880).

### RNA sequencing and data analysis

hEROs treated with or without 2 μg/mL S protein on days 90 and 280 for 48 h were collected, and three independent biological replicates were analyzed by RNA sequencing. Total RNA was extracted using TRIzol according to manufacturer’s instructions, and a NanoDrop and an Agilent 2100 Bionanalyzer (Thermo Fisher Scientific, MA, United States) was used to quantify total RNA. Subsequently, library preparation is performed using Optimal Dual-model mRNA Library Prep Kit (BGI-Shenzhen, China). The mRNA was amplified to obtain the final DNA library, which was then sequenced using PE100/PE150 on the G400/T7/T10 platform (BGI-Shenzhen, China) through the combined probe-anchor ligation technology. The sequencing raw data was filtered using SOAPnuke (v1.5.6). Clean data were aligned to the reference genome using HISAT2 (v2.1.1) and to the reference gene set using Bowtie2 (v2.3.4.3). Gene expression was quantified using RSEM (v1.3.1), and a clustered heatmap of gene expression across different samples was generated using pheatmap (v1.0.8). Differential gene analysis was performed using DESeq2 (v1.4.5) with a significance threshold of Q value < 0.05. To further explore the functions of genes associated with phenotypic changes, we performed GO (https://geneontology.org/) and KEGG (https://www.kegg.jp/) enrichment analyses of differentially expressed genes (DEGs) using Phyper (https://en.wikipedia.org/wiki/Hypergeometric_distribution), based on hypergeometric tests. A threshold of Q value < 0.05 was applied, and genes meeting this criterion were considered significantly enriched among the candidate genes. All analysis was conducted using Dr. Tom.

### Real-time polymerase chain reaction (RT-PCR)

After exposing 90- and 280-day hREOs to the S protein for 48 h, three independent biological replicates (treated with or without the S protein) were collected for RT-PCR. The total RNA extraction and cDNA amplification were performed as described above. Quantitative RT-PCR was conducted using a CFX96 Real-Time PCR System (Bio-Rad, United States) with SYBR^®^ Premix Ex Taq^™^ II (Takara, Japan). Relative expression levels of genes were normalized to GAPDH. The primers used are listed in Table 1.

**Table 1:** The primers are used in RT-PCR

Genes	Forward	Reverse
MYD88	GGCTGCTCTCAACATGCGA	CTGTGTCCGCACGTTCAAGA
TLR4	AGACCTGTCCCTGAACCCTAT	CGATGGACTTCTAAACCAGCCA
LDHA	ATGGCAACTCTAAAGGATCAGC	CCAACCCCAACAACTGTAATCT
HK2	TGCCACCAGACTAAACTAGACG	CCCGTGCCCACAATGAGAC
ID3	GAGAGGCACTCAGCTTAGCC	TCCTTTTGTCGTTGGAGATGAC
TGFβ1	GGCCAGATCCTGTCCAAGC	GTGGGTTTCCACCATTAGCAC
COL1A1	GAGGGCCAAGACGAAGACATC	CAGATCACGTCATCGCACAAC
COL6A3	CATAACCGCTGTGCGGAAAAT	TCATCTAGGGACTTACCACCT
FN1	GAGAATAAGCTGTACCATCGCAA	CGACCACATAGGAAGTCCCAG
ITGA1	GTGCTTATTGGTTCTCCGTTAGT	CACAAGCCAGAAATCCTCCAT
THBS2	GACACGCTGGATCTCACCTAC	GAAGCTGTCTATGAGGTCGCA
DHCR24	CACTGTCTCACTACGTGTCGG	CCAGCCAATGGAGGTCAGC
INSIG1	ATCCAGAGGAATGTCACTCTCTT	AGGGGTACAGTAGGCCAACAA
E2F1	CATCCCAGGAGGTCACTTCTG	GACAACAGCGGTTCTTGCTC
LDLR	ACGGCGTCTCTTCCTATGACA	CCCTTGGTATCCGCAACAGA
GAPDH	CCATGTTCGTCATGGGTGTGA	CATGAGTCCTTCCACGATACCA

### Statistical analysis

Data are presented as means ± SD. All results were analyzed from at least three independent experiments using GraphPad Prism 9 (San Diego, CA). One-way analysis of variance (ANOVA) with Tukey’s multiple comparison test and Student’s t-test were used to evaluate the differences. A *P* -value< 0.05 was considered significant.

## Results

### Generation of hEROs as a model of retinal development

**Fig. 1:**
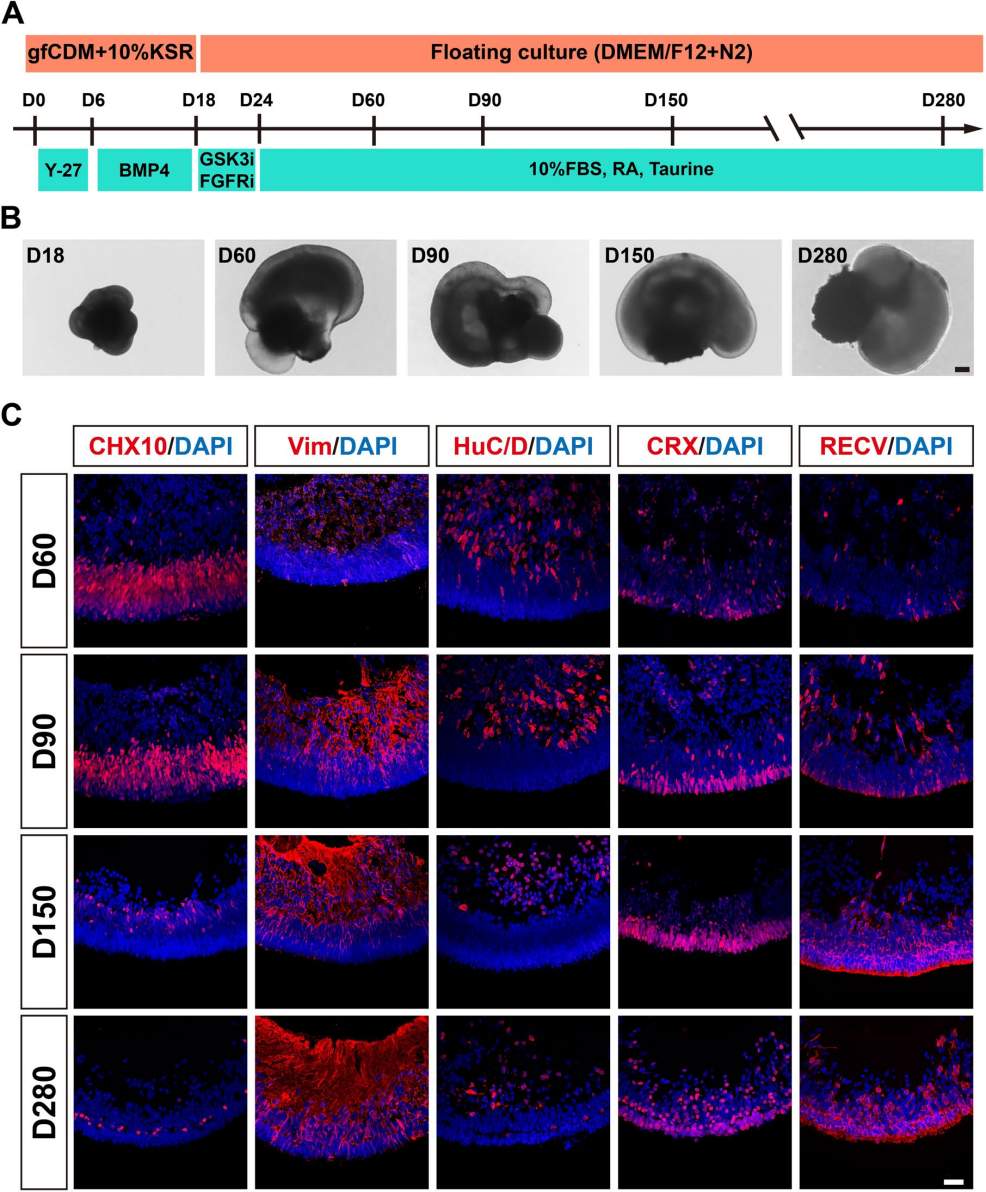
Generation and identification of hEROs in vitro. **A** Schematic of the hEROs self-organization protocol. **B** Bright-field view showing the representative images of hEROs at days 18, 60, 90, 150, and 280. Scale bar, 100 μm. **C** Immunostaining of hEROs at days 18, 60, 90, 150, and 280 for CHX10^+^, Vimentin (Vim)^+^, HuC/D^+^, CRX^+^, and Recoverin (RECV)^+^ cells. Scale bar, 50 μm

By slightly modifying Kuwahara’s inducing protocol [39], we initially aggregated the hESCs into embryoid bodies, and then differentiated into the neural retina (NR) through transient early treatment with BMP4 on day 6. Following 12 days of BMP4 treatment, we switched the retinal organoids to induction-reversal culture media for 6 days to induce retinal pigment epithelium (RPE) differentiation. Subsequently, the media was exchanged for long-term culture medium to establish mature hEROs (Fig. 1A). After being transferred to induction-reversal culture medium, RPE- neural retina structures gradually formed and can be maintained until day 280 (Fig. 1B). To characterize the NR, the retina-specific markers in hEROs were analyzed using immunostaining, including the retinal progenitor marker CHX10, the retinal amacrine/ganglion cell marker HuC/D, Müller cell marker Vimentin, photoreceptor markers CRX and Recoverin, from day 60 to day 280 (Fig. 1C). The results showed that the quantities of CHX10^+^ and HuC/D^+^ cells were relatively high on days 60 and 90 of early differentiation, but declined over time and were located mainly in the inner retinal layer (Fig. 1C). The number of Vimentin^+^, CRX^+^, and Recoverin^+^ cells increased from day 60 to day 280, indicating hEROs continue to develop towards a mature retina-like fate. The pseudo-photoreceptor layer began to form at day 90, as demonstrated by CRX and Recoverin staining (Fig. 1C), indicating the early stage of photoreceptor layer development [40, 41]. By day 280, the outer segment structures of photoreceptor cells had become more prominent, as revealed by Recoverin staining (Fig. 1C), marking the late stage of photoreceptor layer development [40, 41]. Therefore, we selected days 90 and 280 for the S protein exposure study.

### ACE2 and TMPRSS2 were stably expressed in the hEROs during retinal development

**Fig. 2:**
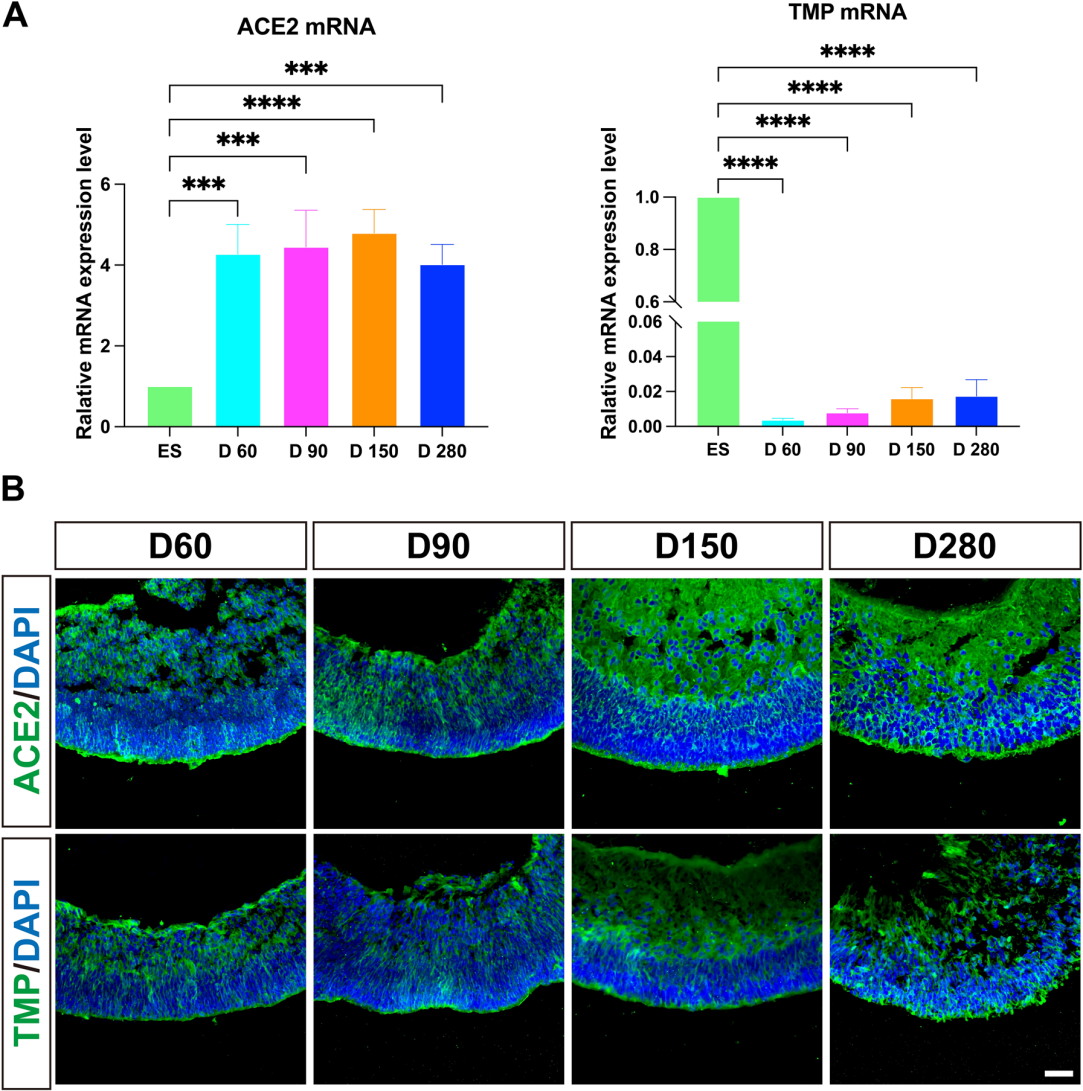
Expression of SARS-CoV-2 receptor in hEROs during embryonic retinal development. **A** The relative mRNA expression of ACE2 and TMPRSS2 (TMP) in hECSs (ES) and hEROs (D60, D90, D150, and D280). **B** Representative images of the expression of ACE2^+^ and TMP^+^ cells at day 60, 90, 150, and 280 by immunofluorescence staining. Scale bar, 50 μm. Data are presented as means ± SD. ***P < 0.001, ****P < 0.0001

ACE2 is a key receptor of SARS-CoV-2 and has been found to be expressed in both human and rodent retinas [42, 43]. The presentation of TMPRSS2, which facilitates viral entry into host cells via ACE2 [15]. Menuchin-Lasowski et al. demonstrated that SARS-CoV-2 infected the retinal ganglion cells and photoreceptors in retinal organoids [33]. However, the expression of ACE2 and TMPRSS2 at different stages of embryonic retinal development is still not clear. Thus, we performed RT-PCR to evaluate the expression of ACE2 and TMPRSS2 in hESCs and hEROs (from day 60 to day280). Compared with hESCs, ACE2 mRNA levels were upregulated in hEROs from day 60 to day 280, while TMPRSS2 mRNA levels were significantly downregulated (Fig. 2A ). There was no significant difference in the expression levels of ACE2 and TMPRSS2 mRNA from day 60 to day 280. In addition, the ACE2^+^ and TMPRSS2^+^ cells were identified by immunostaining from day 60 to day 280 (Fig. 2B). The results showed that ACE2^+^ cells were distributed throughout the NRs, while the number of TMPRSS2^+^ cells were lowly detected in NRs. Taken together, these results demonstrated that ACE2 and TMPRSS2 are expressed in th hEROs during embryonic retina development.

### The influence of S protein exposure on retinal cells in hEROs

**Fig. 3:**
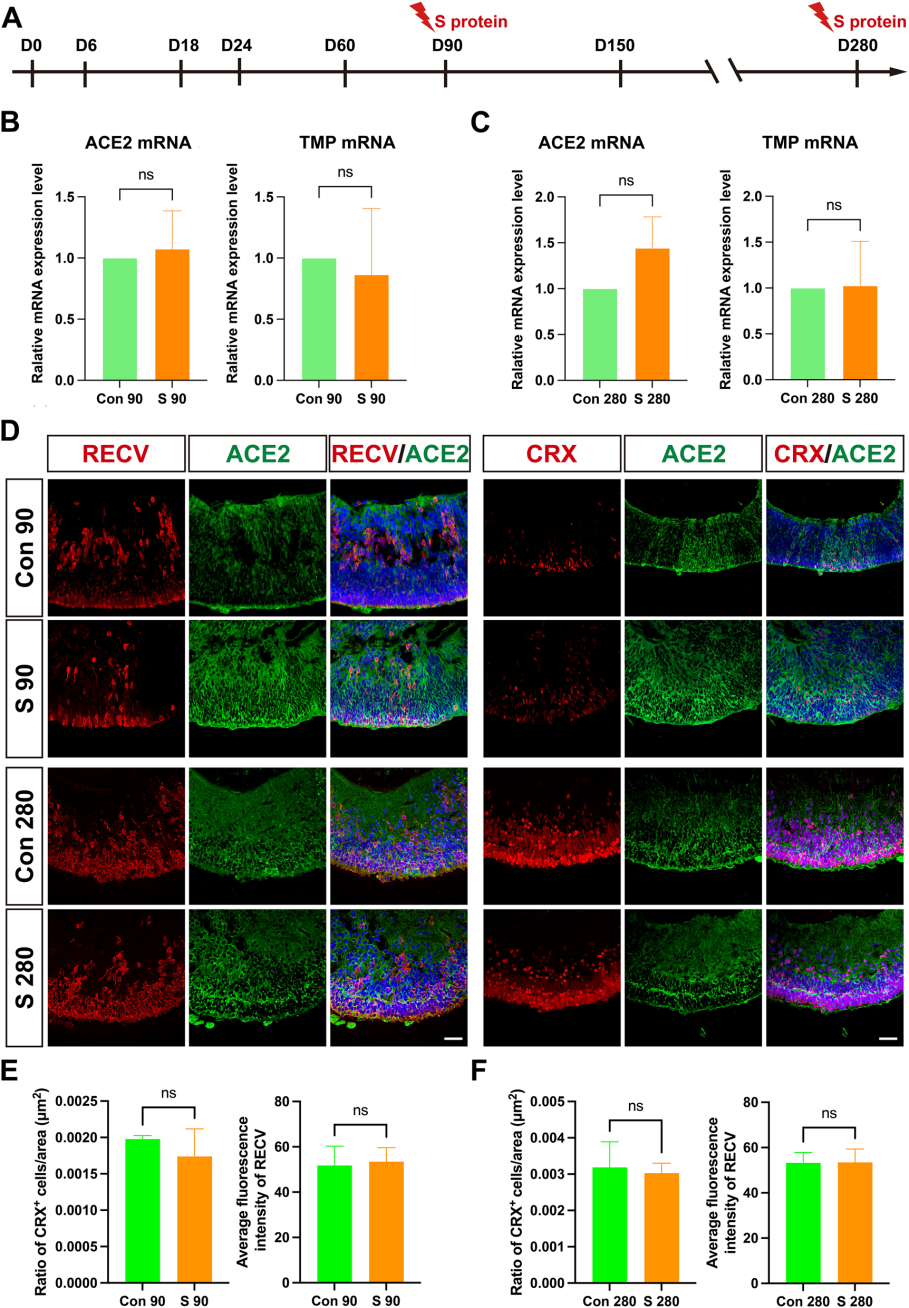
The influence of the SARS-CoV-2 S protein exposure on the development of hEROs. **A** Schematic of S protein treatment of hEROs at different points. **B** The relative mRNA levels of ACE2 and TMP in hEROs at day 90 after S protein exposure, compared to control groups . **C** The relative mRNA levels of ACE2 and TMP in hEROs at day 280 after S protein exposure, compared to control groups . **D** Representative images of the expression of RECV^+^/ACE2^+^ cells and CRX^+^/ACE2^+^ cells in hEROs at days 90 and 280 after S protein exposure. **E** The ratio of CRX-positive cells/ area (μm^2^) and the average fluorescence intensity of RECV- positive cells in 90-day hEROs after S protein exposure, compared to control groups . **F** The ratio of CRX-positive cells/ area (μm^2^) and the average fluorescence intensity of RECV- positive cells in 280-day hEROs after S protein exposure, compared to control groups. Data are presented as means ± SD. Scale bar, 50 μm

To understand the influence of S protein on retinal development, we treated 90-day hEROs with 1, 2, 5, and 10 μg/mL of S protein. It was showed that the mRNA levels of TLR4 and MYD88 were significantly increased with 2 μg/mL of S protein exposure in the RT-PCR test (Fig. S1A), so 2 μg/mL of S protein was selected as the exposure concentration to the hEROs at both 90  and 280 days (Fig. 3A), which mimic the early and late stages of photoreceptor layer development [40, 41]. The treated hEROs were analyzed by RT-PCR to assess the expression of S protein receptors. Compared with the control group, the mRNA levels of ACE2 and TMPRSS2 showed no significant difference after exposure to S protein on days 90 and 280 (Fig. 3B, C). In addition, the thickness of the NRs at days 90 and 280 showed no significant change (Fig. S1B). It was also confirmed via immunostaining that there were no significant changes in the Ki67, Caspase 3, and TUNEL-positive cells after S protein exposure on both days 90 and 280 (Fig. S1 C-E). Furthermore, to identify changes in retinal cells, S protein-treated hEROs on days 90 and 280 were co-stained with ACE2 and retinal cell-type-specific markers (Fig. 3D and Fig. S2). Co-staining of ACE2 with CRX and Recoverin in 90-day and 280-day hEROs indicated the susceptibility of S protein exposure in the photoreceptors of relatively immature or mature hEROs (Fig. 3D). A small number of HuC/D^+^ amacrine/ganglion cells and AP2α^+^ amacrine cells also stained positive for ACE2, indicating that the retinal ganglion cells and amacrine cells are likely susceptible to S protein exposure (Fig. S2A). However, analysis of the ratio of CRX-positive cells and the average fluorescence intensity of Recoverin indicated that short-term exposure to the S protein did not cause significant changes in photoreceptor cells on either days 90 or 280 (Fig. 3E, F). Additionally, analysis of the ratio of HuC/D- and AP2α-positive cells showed no significant alterations in amacrine and ganglion cells following S protein treatment (Fig. S2B, C).

### Transcriptomic analysis of hEROs with S protein exposure

To investigate the effect of S protein exposure on the development of hEROs at transcriptional level, we collected samples from treated and untreated hEROs at days 90 and 280, and subjected them to RNA sequencing. By comparing the 90-day group with the 280-day group, GO biological process (BP) analysis showed that “Visual perception”, “Nervous system development”,  and “Phototransduction” were the top three significantly enriched processes (Fig. S3A). GO cellular component (CC) analysis showed that “Integral component of plasma membrane”, “Photoreceptor outer segment”,  and “Plasma membrane” were the top three significantly enriched components (Fig. S3B). In addition, GO BP-GSEA showed that “Sensory perception of light stimulus” was enriched (Fig. S3C), and GO CC-GSEA showed that “Photoreceptor outer segment” was enriched (Fig. S3D). These findings imply that hEROs in this study can mimic retinal development, as well as the maturation of photoreceptors and the visual perception system over time, thus representing an effective model for studying the effects of S protein exposure.

**Fig. 4:**
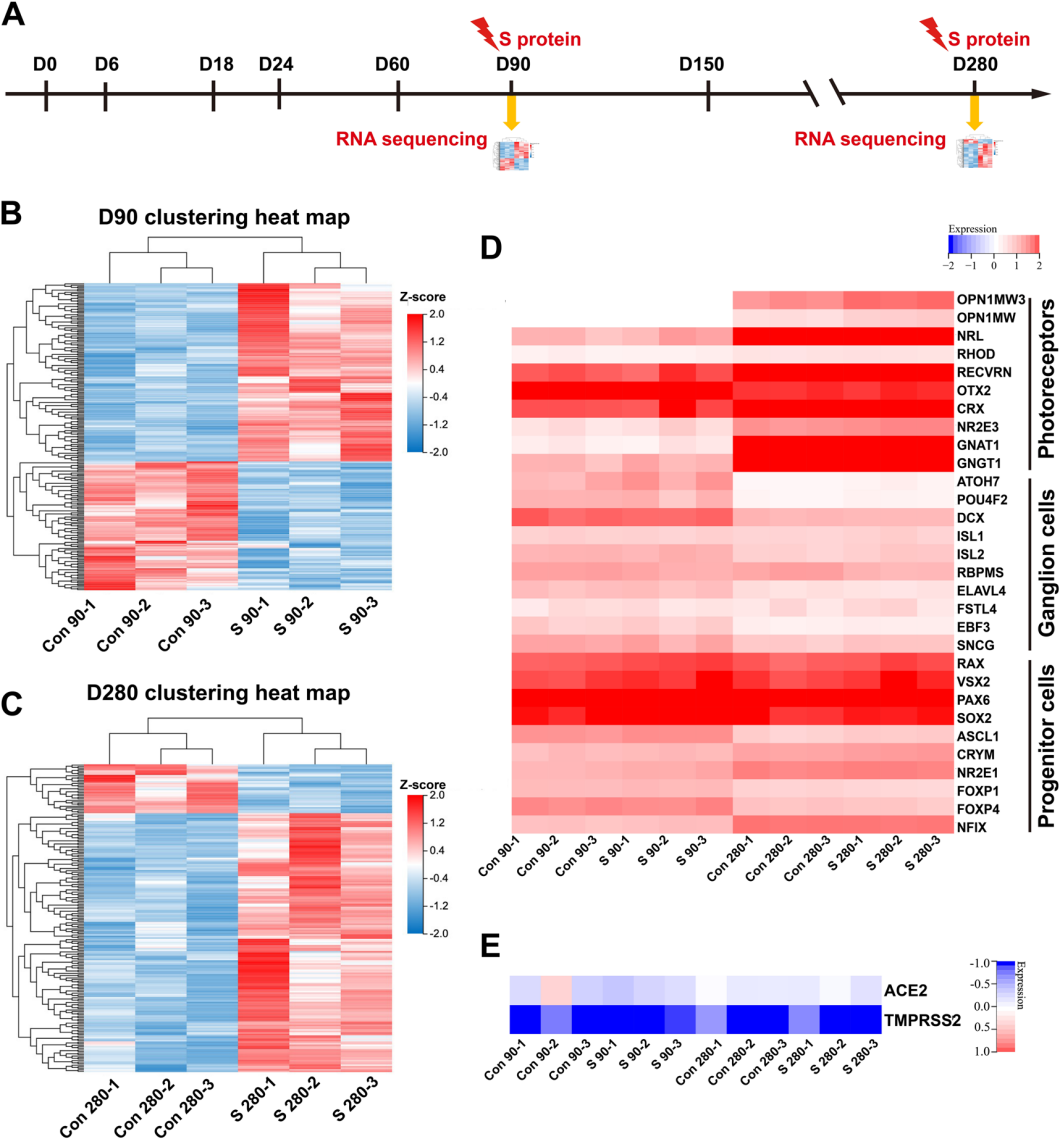
RNA sequencing analysis of the influence of S protein exposure on hEROs on days 90 and 280. **A** Schematic of the RNA sequencing protocol for S protein treatment of hEROs. **B** The hierarchical clustering heatmap of the control and S protein treatment groups on day 90. **C** The hierarchical clustering heatmap of the control and S protein treatment groups on day 280. **D** The hierarchical clustering heatmap of genes associated with photoreceptors, retinal ganglion cells, and progenitor cells in hEROs on days 90 and 280 for both groups, respectively. **E** The hierarchical clustering heatmap showed the expression of the SARS-CoV-2 receptor ACE2 and TMPRSS2 in hEROs on days 90 and 280 for both groups, respectively

Previous studies have demonstrated that SARS-CoV-2 can infect neurons and neural progenitors [44]. Next, we tested the effects of S protein exposure on hEROs during retinal development by comparing the transcriptome of non-treated hEROs with that of hEROs treated with S protein for 48 h on days 90 and 280 (Fig. 4A). The clustering heat map showed that the three biological replicates on days 90 and 280 after exposure to S protein were homogeneous, indicating the repeatability and reliability of the experimental data (Fig. 4B, C). To test the influence of S protein on retinal cells associated with photoreceptors, retinal ganglion cells, and progenitor cells, we integrated and analyzed the count matrices of non-treated and treated hEROs , and used a curated set of known gene markers to annotate the three cell groups. The gene heat map for non-treated hEROs on days 90 and 280 reflected the maturation of photoreceptors and the reduction in retinal ganglion cells (Fig. 4D). Notably, there was nearly no difference between non-treated and treated hEROs on days 90 and 280 for the three cell types (Fig. 4D). Consistent with the RT-PCR results shown in Fig. 3B and C, transcriptome analysis also revealed no significant difference in the mRNA expression levels of ACE2 and TMPRSS2 (Fig. 4E). Taken together, these results demonstrated that there was no significant influence on retinal cell types after short-term S protein exposure at both early and late stages of retinal development.

### Different effects of S protein on gene expression during retinal development

**Fig. 5:**
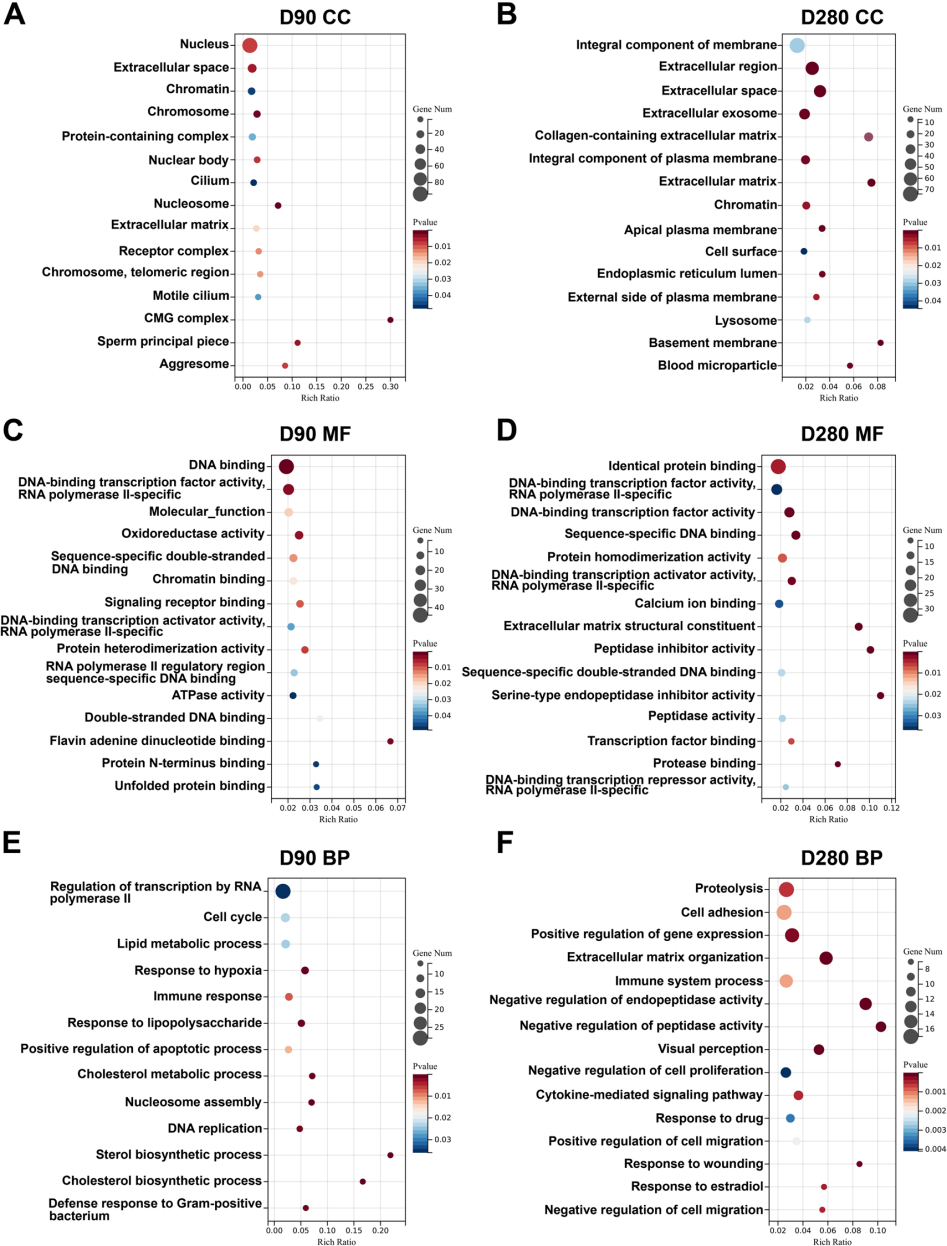
S protein exposure exhibits different effects on retinal development at different stages. **A** Enriched GO terms in CC category in  90-day hEROs after S protein exposure, compared to control groups. The statistically significant enriched GO terms were ranked by gene numbers. **B** Enriched GO terms in CCs category in 280-day hEROs after S protein exposure, compared to control groups. The statistically significant enriched GO terms were ranked by gene numbers. **C** Enriched GO terms in MF category in 90-day hEROs after S protein exposure, compared to control groups. The statistically significant enriched GO terms were ranked by gene numbers. **D** Enriched GO terms in MF category in 280-day hEROs after S protein exposure, compared to control groups. The statistically significant enriched GO terms were ranked by gene numbers. **E** Enriched GO terms in BP category in  90-day hEROs after S protein exposure, compared to control groups. The statistically significant enriched GO terms were ranked by gene numbers. **F** Enriched GO terms in BP category in 280-day hEROs after S protein exposure, compared to control groups. The statistically significant enriched GO terms were ranked by gene numbers

To gain further insights into the processes occurring in the hEROs upon S protein treatment, GO analysis, including CC, BP, and molecular function (MF) on days 90 and 280 of differentiation was performed. The top 5 most enriched CC categories in the analysis of 90-day non-treated and treated hEROs were highly related to cell nucleus, including “Nucleus”, “Chromatin” and “Chromosome”, whereas in 280-day hEROs, the DEGs were more closely associated with membrane and matrix components, such as “Integral component of membrane”, “Extracellular region”, “Extracellular space”, “Extracellular exosome” and “Collagen containing extracellular matrix” (Fig. 5A, B). GO MF analysis revealed that the pathways enriched by DEGs between non-treated and treated hEROs at 90 days were primarily associated with DNA binding, while those at 280 days were mainly related to protein recognition (Fig. 5C, D). GO BP analysis indicated that DEGs were enriched in immune-related pathways at both 90 and 280 days (Fig. 5E, F). At 90 days, the main enriched pathways were associated with nucleic acid transcription, cell cycle, and metabolic processes, while at 280 days, the primary enrichments were in cell adhesion, ECM, and cell migration pathways. Except for the top 5 of the GO analysis, the results also showed enrichment of “Motile cilium”, “Response to lipopolysaccharide”, and “Cholesterol metabolic process” in 90-day hEROs, while “Calcium ion binding”, “Visual perception”, and “Positive regulation of cell migration” were enriched in 280-day hEROs (Fig. 5F). These results suggest that S protein exposure exhibits different effects on the differentiation of retina, which mostly involves processes occurring in nucleus, lipid metabolic process, immune response at the early stages, while the processes associated with cell membrane components, immune system process, and cell migration at the late stages of retinal development.

### S protein exposure disrupted the gene expression of hEROs at different stages of retinal development

**Fig. 6:**
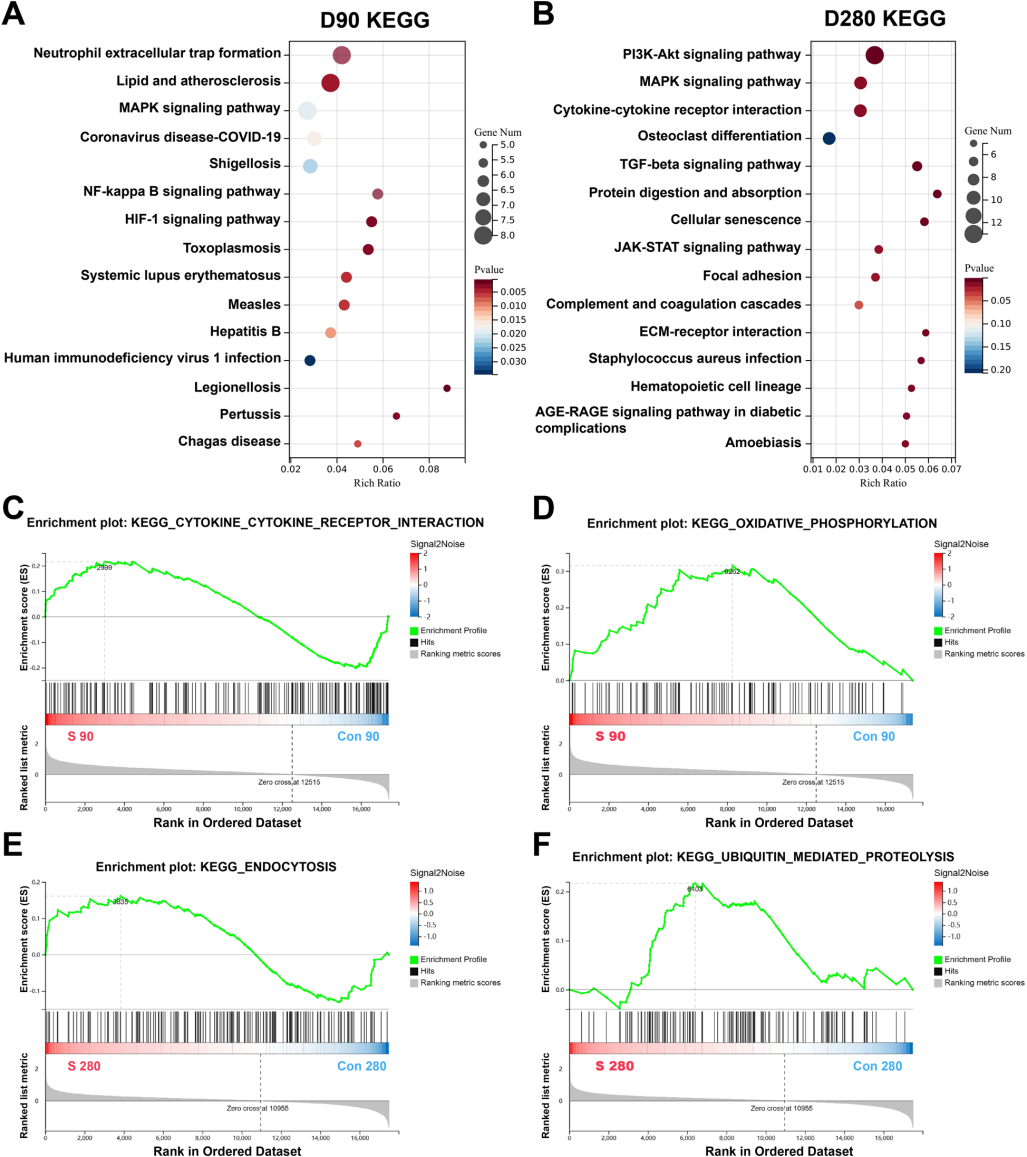
S protein exposure disrupts retinal development at different stages. **A** The enrichment of KEGG pathways in 90-day hEROs after S protein exposure, compared to control groups. The statistically significant enriched KEGG pathways were ranked by gene numbers. **B** The enrichment of KEGG pathways in 280-day hEROs after S protein exposure, compared to control groups. The statistically significant enriched KEGG pathways were ranked by gene numbers. C, **D** GSEA analysis shows the enrichment of gene sets associated with cytokine receptor interaction and oxidative phosphorylation in 90-day hEROs after S protein exposure, compared to control groups. **E**, **F** GSEA analysis shows the enrichment of gene sets associated with endocytosis and ubiquitin mediated proteolysis in 280-day hEROs after S protein exposure, compared to control groups

To further investigate the biomolecular pathogenesis of S protein exposure, we performed KEGG enrichment analysis. The KEGG pathway analysis showed the DEGs were closely related to “Neutrophil extracellular trap formation”, “Lipid and aterosclerosis”, “MAPK signaling pathway”, “Coronavirus disease-COVID-19”, “Shigellosis”, “NF-kappa B signaling pathway”, and “HIF-1 signaling pathway” that were mainly enriched at day 90 exposed to S protein, compared witht the control group (Fig. 6A). In addition, the KEGG enrichment analysis of 280-day hEROs treated with S protein showed “PI3K-Akt signaling pathway”, MAPK signaling pathway, “Cytokine-cytokine receptor interaction”, “TGF-β signaling pathway”, “Protein digestion and absorption”, “Cellular senescence”, “JAK-STAT signaling pathway”, and “ECM-receptor interaction” were mainly associated with the DEGs (Fig. 6B). The GSEA analysis revealed that “Cytokine-cytokine receptor interaction” and “Oxidative phosphorylation” were enriched in 90-day hEROs after S protein (Fig. 6C, D). Furthermore, “Endocytosis” and “Ubiquitin mediate proteolysis” were identified by GSEA analysis (Fig. 6E, F). In all, the KEGG pathway analysis of DEGs also indicated that genes related to inflammatory pathways were enriched at both time points. Additionally , metabolic pathways were predominantly enriched at 90 days, while pathways related to the ECM were enriched at 280 days.

**Fig. 7:**
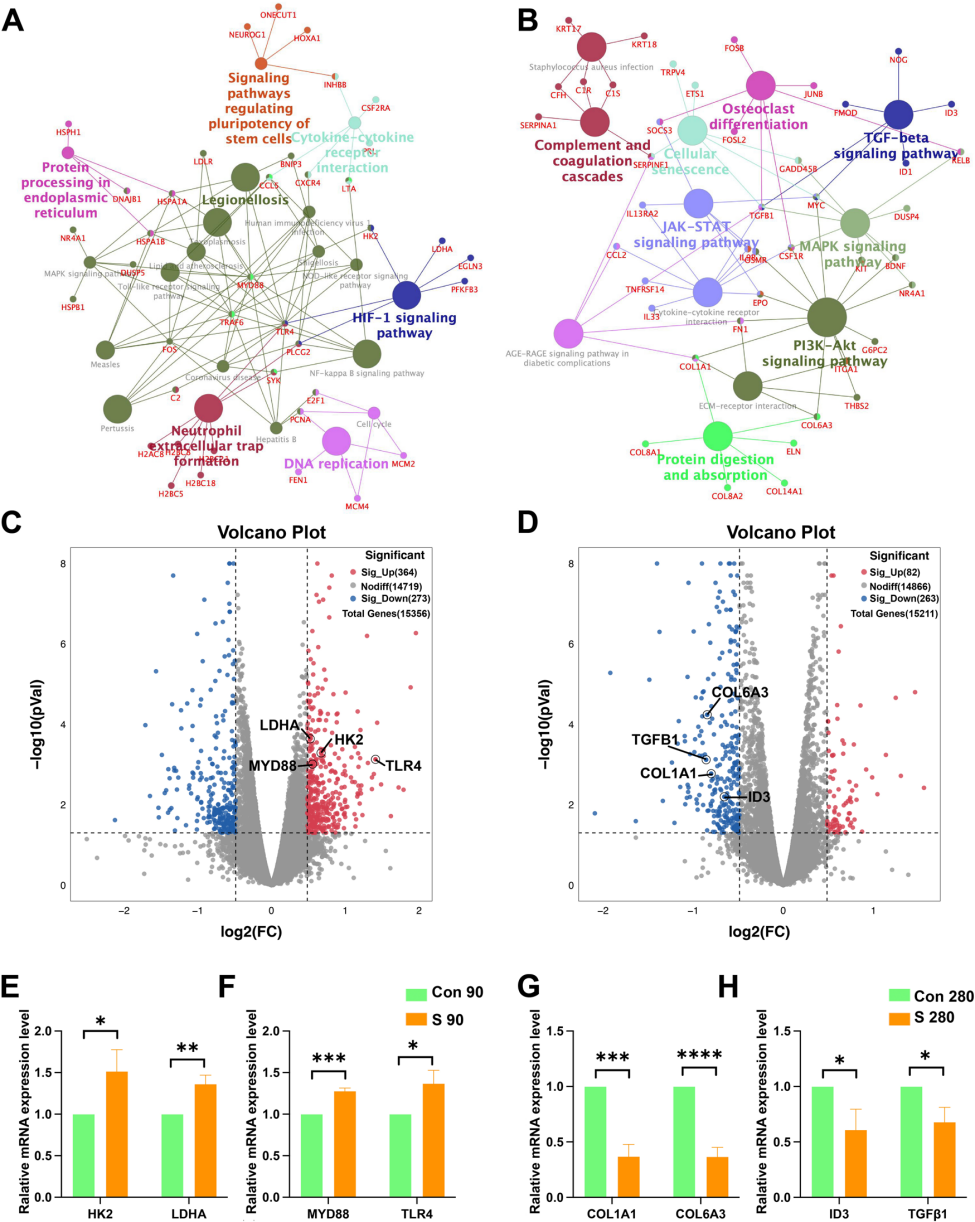
S protein exposure shows the influences of retinal development at the molecular levels. **A** The  KEGG pathway signaling interaction network of the DEGs in 90-day hEROs after S protein exposure, compared to control groups. **B** The KEGG pathway signaling interaction network of the DEGs in 280-day hEROs after S protein exposure, compared to control groups. **C** Volcano plot shows the DEGs of S protein exposure in hEROs at day 90, compared to control groups. **D** Volcano plot shows the DEGs of S protein exposure in hEROs at day 280, compared to control groups. **E**, **F** The relative mRNA expression level of HK2, LDHA, MYD88, and TLR4 in 90-day hEROs  after S protein exposure, compared to control groups. **G**, **H** The relative mRNA expression level of COL1A1, COL6A3, ID3, and TGFβ1 in 280-day hEROs  after S protein exposure, compared to control groups. Data are presented as means ± SD. *P < 0.05, **P < 0.01, ***P < 0.001, ****P < 0.0001

Based on KEGG signaling pathway analysis, gene-pathway interaction mapping showed the HIF-1 signaling pathway was enriched with LDHA and HK2, while the coronavirus disease and NF-kappa B signaling pathway were co-enriched with TLR4 and MYD88 in 90-day hEROs exposed to S protein (Fig. 7A). But to 280-day hEROs that treated with S protein, the gene-pathway interaction mapping showed the TGFβ signaling pathway genes ID3 and TGFβ were enriched, which was directly connected to MAPK signaling pathway, cytokine-cytokine receptor interaction, JAK-STAT signaling pathway, and cell senescence (Fig. 7B). In addition, COL1A1 and COL6A3 that were related to ECM-receptor interaction, protein digestion and absorption, and PI3K-Akt signaling pathway were also enriched in the network (Fig. 7B). The volcano plot and RT-PCR analysis revealed that glycolysis-related genes LDHA and HK2, along with inflammatory genes MYD88 and TLR4, were upregulated in 90-day treated hEROs compared to the control group, indicating that S protein treatment activated metabolic and inflammatory pathways at the early stages of retinal development (Fig. 7C, E, F). Meanwhile, the mRNA expression levels of genes involved in “lipid metabolism process”, including DHCR24, LDLR, and INSIG1, were significantly downregulated, while E2F1 was upregulated in 90-day hEROs (Fig. S4A). When compared to 280-day hEROs exposed to the S protein, the volcano plot and RT-PCR analysis showed that the anti-inflammatory genes TGFβ1 and ID3, as well as ECM-related genes COL1A1 and COL6A3, were downregulated in the S protein group, demonstrating that the S protein exposure  affected inflammation and the ECM at the late stages of retinal development (Fig. 7D, G, H). In addition, other “ECM-receptor interaction” genes, including FN1, ITGA1, and THBS2, were also demonstrated to be downregulated by RT-PCR (Fig. S4B). Taken together, these results revealed that S protein exposure differentially disrupted the gene expression of hEROs at different stages of retinal development.

## Discussion

Pregnant women are a high-risk group for SARS-CoV-2 infection, with potential viral invasion occurring in both the placenta and embryo [4–6]. However, the influence of SARS-CoV-2 infection on retinal development and the underlying mechanisms are still elusive. Here, we utilized hEROs to investigate the impact of the S protein on the retina at different developmental stages (90 days and 280 days), which is crucial for SARS-CoV-2 invasion and pathogenesis. It was observed that short-term S protein exposure did not alter the expression of SARS-CoV-2 receptor ACE2 and TMPRSS2, nor did it affect the retinal cell number or morphology. Interestingly, transcriptomic analysis revealed that short-term S protein exposure affected nuclear composition during the early developmental stage and exerted an effect on cell membrane composition during the late developmental stage. In addition, RNA sequencing data also showed S protein induced an inflammatory response in both the early and later stage of hEROs development. Notably, hEROs exposed to the S protein in the early stage primarily affected lipid-associated metabolic pathways, while those in the late developmental stages mainly impacted the ECM.

Clinical data on the impact of maternal COVID-19 infection on embryonic retinal development remain limited. Currently, there is scarce direct research on the effects of SARS-CoV-2 on embryonic retinal development, with most studies focusing on placental function and fetal brain development [45, 46]. A study suggests that infants born to mothers with COVID-19 exposure might affect early newborn brain development, characterized by increased cortical gray matter volume and accelerated sulcal depth in the frontal lobe [45]. In a study screening for ocular manifestations in newborns with maternal COVID-19 infection history, external eye examinations and binocular indirect ophthalmoscopy were conducted. Although direct COVID-19 test results for these newborns were negative, one case exhibited venous congestion and vascular tortuosity, while seven cases showed retinal hemorrhages. No significant ocular abnormalities were detected in the six newborns who tested positive for COVID-19 [47]. Moreover, severe ocular malformations, including unilateral microphthalmia, optic nerve hypoplasia, and congenital retinopathy, have been observed in association with maternal SARS-CoV-2 infection during the fifth and sixth weeks of embryonic development [48]. Notably, the course of SARS-CoV-2 infection, viral virulence, and the developmental stage at time of infection all might influence ocular developmental abnormalities. Additionally, the limited neonatal morphological and functional assessment methods, as well as the timing of evaluation, could also affect outcome interpretation. In this study, hEROs treated with the spike protein exhibited alterations in inflammation-related genes, consistent with the  pathogenic characteristics of the virus. Interestingly, we found that exposure to the S protein during early photoreceptor layer development primarily affected lipid-associated metabolic pathways, while late-stage exposure mainly affected the ECM. Although changes were primarily observed at the gene expression level, the correlation with clinical manifestations requires further investigation. Given the known neurotropic properties of SARS-CoV-2, it is plausible that viral exposure during pregnancy could influence retinal neurodevelopment, warranting further clinical and epidemiological studies, as well as an urgent need to establish optimized in vitro models to study the pathogenic mechanisms and effects.

Accumulating evidence demonstrated that ACE2, the key viral entry receptor for SARS-CoV-2, has been detected on the ocular surface and in the retina [23, 32, 49]. In addition, several reports identified SARS-CoV-2 RNA in retinal biopsies from COVID-19 positive patients, while different retinal anomalies were observed in these patients [9, 50–52]. However, a study reported that the failure to isolate the virus or detect any SARS-CoV-2 S protein from SARS-CoV-2 RNA-positive human retinal biopsies, suggesting that SARS-CoV-2 can infect but not replicate in retinal cells [50]. Using retinal organoids, another study revealed that SARS-CoV-2 can infect and replicate in retinal cells through the ACE2 pathway [33]. Currently, there is a lack of conclusive evidence regarding the infection of SARS-CoV-2 in the human retina, and the impact of SARS-CoV-2 infection on retinal structures remains unclear. Interestingly, compared with human embryonic stem cells, our results demonstrated that the expression level of ACE2 mRNA was significantly increased in hEROs from days 60 to 280, while TMPRSS2 mRNA decreased. The expression levels of ACE2 and TMPRSS2 are similar to those observed on the ocular surface during human embryonic development [22]. After exposure to S protein, the mRNA levels of ACE2 and TMPRSS2 showed no significant change at day 90 and day 280 in hEROs, suggesting that the S protein treated retina does not require the upregulation of ACE2. These results are inconsistent with previous findings of short-term S protein exposure, where ACE2 expression was significantly altered [16, 53–55]. However, previous reports have shown inconsistent changes in ACE2 and TMPRSS2 receptors following S protein exposure, with some studies indicating no change, while others have reported either increased or decreased levels [56–61]. These results may be caused by the differences between the present study and previous reports in the duration or intensity of S protein exposure and tissue heterogeneity.

Currently, inflammation is ubiquitous in SARS-CoV-2-triggered eye diseases, such as dry eye, conjunctivitis, and retinal degenerative diseases [33, 62]. We therefore tested the inflammation activation at both the early and later stages of hEROs. The inflammatory genes involved in MYD88 and TLR4 were increased in day-90 hEROs, while the anti-inflammatory genes TGFβ and ID3 were decreased in the day-280 hEROs, suggesting that the immunosuppressive response of the retina may rescue the SARS-CoV-2-induced eye diseases. In addition, the transcriptomic analysis showed that younger hEROs appeared to influence nuclear composition, while older hEROs seemed to primarily affect cell membrane composition (Fig. 5). This suggests that SARS-CoV-2 infection at different stages of retinal development has varying effects.

Previous studies have revealed that lipid metabolism plays a central role in COVID-19 diseases [63, 64]. Wu et al. reported that dysregulations in lipid metabolism led to exceedingly drastic changes in fatal SARS-CoV-2 positive patients compared to survivors [63]. After infection with SARS-CoV-2 virus, the expression of apolipoproteins genes associated with macrophage functions was decreased [64]. The steroid hormones, which have the function of stimulating the activity of macrophages, were accumulated in COVID-19 positive patients [64, 65]. For the retina, it has been demonstrated that disturbing the lipid metabolism leads to impaired cone function [66]. Li et al. revealed that exposure to polybrominated diphenyl ethers to retinal organoids led to significant changes in purine and glutathione metabolism [67]. In addition, analyzing the transcriptome dynamics of mouse-derived retinal organoid development reveals that lipid metabolism plays an important biological role during retinal development [68]. Our results showed that lipid metabolism was altered in day-90 hEROs. The lipid and atherosclerosis, Coronavirus disease-COVID-19, NF-kappa B signaling pathway, and HIF-1 signaling pathway were enriched in the early stages of retinal development. Furthermore, the lipid metabolism genes HK2 and LDHA were upregulated in the 90-day hEROs. These results suggested that exposure to SARS-CoV-2 at the early stages of retinal development primarily effects lipid metabolism.

The ECM, a non-cellular 3D macromolecular network mainly composed of proteoglycans, collagens, elastin, fibronectin, laminins, functions to regulate diverse cellular processes. COVID-19 is associated with abnormal expression of the ECM, which is involved in recruiting inflammatory cells and interfering with target organ metabolism [69]. After infection with the SARS-CoV-2 virus, COVID-19 patients showed a loss of many core ECM proteins, such as collagens, proteoglycans, and glycoproteins, resulting in damage to the structure and function of lungs [70]. In addition, the transcriptome landscape of human airway epithelium treated with SARS-CoV-2 showed deregulation of genes encoding intercellular communication and adhesion proteins [71]. Thus, the ECM is associated with SARS-CoV-2 infection and is involved in its pathogenesis. The neural ECM proteins are necessary for retinal function, synaptic signaling, and visual motion processing [72]. During ischemic injury and optic nerve neurodegeneration, ECM constituents, such as laminin, chondroitin sulfate proteoglycans aggrecan, and glycoproteins fibronectin, were dysregulated [73]. In addition, the study conducted by Reinhard et al. provided evidence that ECM remodeling occurs in the retina and optic nerve of glaucoma mouse model [74]. In our work, KEGG pathway enrichment analysis showed that the PI3K-AKT signaling pathway, cytokine-cytokine receptor interaction, TGFβ signaling pathway, focal adhesion, and ECM-receptor interaction were enriched. The KEGG-GSEA analysis showed that endocytosis and ubiquitin mediate proteolysis were associated with S protein-treated hEROs at the later stages of retinal development. The expression levels of ECM genes COL1A1 and COL6A3 also increased in 280-day hEROs. These results indicated that the deregulation of ECM components leads to disruption of the retinal development at later stages.

In present study, the use of retinal organoids, while providing a controlled and simplified model, has emerged as a valuable in vitro model for studying retinal development and diseases. However, there are still several limitations to using in vitro organoids to model human retinal development, as this model does not fully capture the complexity of human tissue in vivo, and certain aspects of retinal pathology may not be accurately represented. Firstly, the lack of an immune system may impede the understanding of the host’s antiviral response and inflammatory pathways following viral infection. Secondly, the absence of a vascular system in retinal organoids, affecting their survival and maturity, as well as research on pathological mechanisms related to vasculature. Thirdly, the heterogeneity in retinal organoid cultures between different laboratories limits the accuracy of modeling. These limitations hinder the study of immune-mediated retinal damage, modeling viral entry through the bloodstream, and the reproducibility of results across different laboratories, which may affect the expression of viral entry receptors like ACE2 and TMPRSS2. Despite these limitations, retinal organoids remain a useful model for dissecting cell-specific viral responses and elucidating the direct impact of S protein exposure on retinal cells in a controlled environment. Moreover, there is currently no direct evidence indicating the specific concentrations of the spike protein in pregnant women or developing fetuses during maternal SARS-CoV-2 infection, which poses certain challenges for in vitro experimental simulations. However, numerous studies have utilized varying concentrations of the spike protein in vitro to explore the underlying effects and mechanism of SARS-CoV-2 infection, which provides us with valuable references. Despite the limitations in experimental design, our study provides valuable insights into the potential effects of SARS-CoV-2 spike protein exposure on embryonic retinal development and the related molecular mechanisms. However, further studies using more physiologically relevant models are needed to fully understand the implications of SARS-CoV-2 on retinal and central nervous system pathology. In addition, this study has identified that the regulatory processes associated with lipid metabolism and ECM disruption were probably involved in the developmental impairments induced by SARS-CoV-2 infection. Further in-depth exploration of specific signaling pathways or upstream and downstream pathways will undoubtedly enhance the mechanistic depth of related research. Investigating whether direct modulation of these pathways can rescue the observed disruptions in retinal development will be an important direction for future studies.

## Conclusion

In conclusion, this work revealed that short-term exposure of hEROs to the S protein primarily led to transcriptional alterations, characterized by an inflammatory response-related signature during both the early and advanced stages of retinal development. Additionally, transcriptomic analysis showed that early exposure of S protein primarily affected the cell nucleus and the lipid metabolism-related gene expression. In contrast, at the later stages of embryonic retinal development, S protein exposure mainly influenced gene expression related to ECM and cell membrane composition. These findings provide novel insights into the effects of the S protein during SARS-CoV-2 infection at different stages of retinal development, analyzed at both cellular and molecular levels, which helps in understanding the mechanisms of COVID-19-related retinal pathologies during retinal development.

## Supplementary Information

Supplementary material 1: Fig. 1 The influence of S protein exposure on the development of retinal in human retinal organoids. (A) The mRNA levels of TLR4 and MYD88 in 90-day hEROs exposed to 1, 2, 5, and 10 μg/mL S protein. (B) The thickness of NRs in day-90 and 280 hEROs exposed to 2 μg/mL S protein. (C) Representative images of the expression of Ki67^+^, Caspase 3 (Casp 3)^+^, and TUNEL^+^ cells in hEROs at day 90 and 280 exposed to 2 μg/mL S protein. Scale bar, 50 μm. (D) The ratio of Ki67, Casp 3, and TUNEL-positive cells/ area (μm^2^) in 90-day hEROs after S protein exposure, compared to control groups. (E) The ratio of Ki67, Casp 3, and TUNEL-positive cells/ area (μm2) in 280-day hEROs after S protein exposure, compared to control groups. Data are presented as means ± SD. *P<0.05, **P<0.001, **** *P*<0.0001. Fig. 2 The influence of S protein exposure on the retinal amacrine and ganglion cells in hEROs. (A) Representative images of the expression of AP2^+^/ACE2^+^ and HuC/D^+^/ACE2^+^ cells in hEROs at day 90 and 280 in both two groups. Scale bar, 50 μm. (B) The ratio of AP2 and HuC/D -positive cells/ area (μm^2^) in 90-day hEROs after S protein exposure, compared to control groups. (C) The ratio of AP2 and HuC/D -positive cells/ area (μm^2^) in 280-day hEROs after S protein exposure, compared to control groups. Data are presented as means ± SD. Fig. 3 Transcriptome analysis for the development of hEROs. (A) Enriched GO terms in the BP category in 280-day hEROs, compared to 90-day hEROs. The statistically significant enriched GO terms were ranked by gene numbers. (B) Enriched GO termss in CC category in 280-day hEROs, compared to 90-day hEROs. The statistically significant enriched GO terms were ranked by gene numbers. (C) GSEA analysis showed the enrichment of gene sets associated with sensory perception of light stimulus in 280-day hEROs, compared to 90-day hEROs. (D) GSEA analysis shows the enrichment of gene sets associated with photoreceptor outer segment in 280-day hEROs, compared to 90-day hEROs. Fig. 4 Changes of mRNA levels of the genes related to lipid metabolism and ECM-receptor interaction in 90-day and 280-day hEROs after short-term S protein exposure, compared to control groups. (A) The relative mRNA levels of lipid metabolism-related genes DHCR24, LDLR, INSIG1, and E2F1 in 90-day hEROs after short-term spike protein exposure, compared to control groups. (B) The mRNA expression levels of ECM-receptor interaction-related genes FN1, ITGA1, and THBS2 in 280-day hEROs after short-term spike protein exposure, compared to control groups. Data are presented as means ± SD. ***P<0.001, **** *P*<0.0001.

## Data Availability

All data are available in the main text or the supplementary materials. The raw data of RNA sequencing are available in the national genomics data center of China, accession number HRA008904.

## Abbreviations

ACE2: Angiotensin converting enzyme 2
BP: Biological process
BMP4: Bone morphogenetic protein 4
CC: Cellular component
CNS: Central nervous system
COVID-19: Coronavirus disease 2019
DEGs: Differentially expressed genes
ECM: Extracellular matrix
GO: Gene ontology
GSEA: Gene set enrichment analysis
hEROs: Human embryonic stem cell-derived retinal organoids
hPSC: Human pluripotent stem cell
KEGG: Kyoto encyclopedia of genes and genomes
MF: Molecular function
NR: Neural retina
ANOVA: One-way analysis of variance
OCT: Optimal cutting temperature
RT-PCR: Real-time polymerase chain reaction
RPE: Retinal pigment epithelium
SARS-CoV-2: Severe acute respiratory syndrome coronavirus 2
S: Spike
TMPRSS2: Transmembrane serine protease 2
