# Supplementary material for: The influence of SARS-CoV-2 spike protein exposure on retinal development in the human retinal organoids
Source: Cell Biosci. 2025 Apr 11;15:43. doi: 10.1186/s13578-025-01383-0 (PMC11987193; doi:10.1186/s13578-025-01383-0)
Supplement: Supplementary file 1 — Supplementary material 1: Fig. 1 The influence of S protein exposure on the development of retinal in human retinal organoids. (A) The mRNA levels of TLR4 and MYD88 in 90-day hEROs exposed to 1, 2, 5, and 10 μg/mL S protein. (B) The thickness of NRs in day-90 and 280 hEROs exposed to 2 μg/mL S protein. (C) Representative images of the expression of Ki67+, Caspase 3 (Casp 3)+, and TUNEL+ cells in hEROs at day 90 and 280 exposed to 2 μg/mL S protein. Scale bar, 50 μm. (D) The ratio of Ki67, Casp 3, and TUNEL-positive cells/ area (μm2) in 90-day hEROs after S protein exposure, compared to control groups. (E) The ratio of Ki67, Casp 3, and TUNEL-positive cells/ area (μm2) in 280-day hEROs after S protein exposure, compared to control groups. Data are presented as means ± SD. *P<0.05, **P<0.001, **** P<0.0001. Fig. 2 The influence of S protein exposure on the retinal amacrine and ganglion cells in hEROs. (A) Representative images of the expression of AP2+/ACE2+ and HuC/D+/ACE2+ cells in hEROs at day 90 and 280 in both two groups. Scale bar, 50 μm. (B) The ratio of AP2 and HuC/D -positive cells/ area (μm2) in 90-day hEROs after S protein exposure, compared to control groups. (C) The ratio of AP2 and HuC/D -positive cells/ area (μm2) in 280-day hEROs after S protein exposure, compared to control groups. Data are presented as means ± SD. Fig. 3 Transcriptome analysis for the development of hEROs. (A) Enriched GO terms in the BP category in 280-day hEROs, compared to 90-day hEROs. The statistically significant enriched GO terms were ranked by gene numbers. (B) Enriched GO termss in CC category in 280-day hEROs, compared to 90-day hEROs. The statistically significant enriched GO terms were ranked by gene numbers. (C) GSEA analysis showed the enrichment of gene sets associated with sensory perception of light stimulus in 280-day hEROs, compared to 90-day hEROs. (D) GSEA analysis shows the enrichment of gene sets associated with photoreceptor outer segment in 280-day hEROs, com [file 13578_2025_1383_MOESM1_ESM.docx]

**Supplement Fig. 1** The influence of S protein exposure on the development of retinal in human retinal organoids. (A) The mRNA levels of TLR4 and MYD88 in 90-day hEROs exposed to 1, 2, 5, and 10 μg/mL S protein. (B) The thickness of NRs in day-90 and 280 hEROs exposed to 2 μg/mL S protein. (C) Representative images of the expression of Ki67^+^, Caspase 3 (Casp 3) ^+^, and TUNEL^+^ cells in hEROs at day 90 and 280 exposed to 2 μg/mL S protein. Scale bar, 50 μm. (D) The ratio of Ki67, Casp 3, and TUNEL-positive cells/ area (μm^2^) in 90-day hEROs after S protein exposure, compared to control groups. (E) The ratio of Ki67, Casp 3, and TUNEL-positive cells/ area (μm2) in 280-day hEROs after S protein exposure, compared tto control groups. Data are presented as means ± SD. **P*<0.05, ***P*<0.001, **** *P*<0.0001.





**Supplement Fig. 2** The influence of S protein exposure on the retinal amacrine and ganglion cells in hEROs. (A) Representative images of the expression of AP2^+^/ACE2^+^ and HuC/D^+^/ACE2^+^ cells in hEROs at day 90 and 280 in both two groups. Scale bar, 50 μm. (B) The ratio of AP2 and HuC/D -positive cells/ area (μm^2^) in 90-day hEROs after S protein exposure, compared to control groups. (C) The ratio of AP2 and HuC/D -positive cells/ area (μm^2^) in 280-day hEROs after S protein exposure, compared to control groups. Data are presented as means ± SD.

**
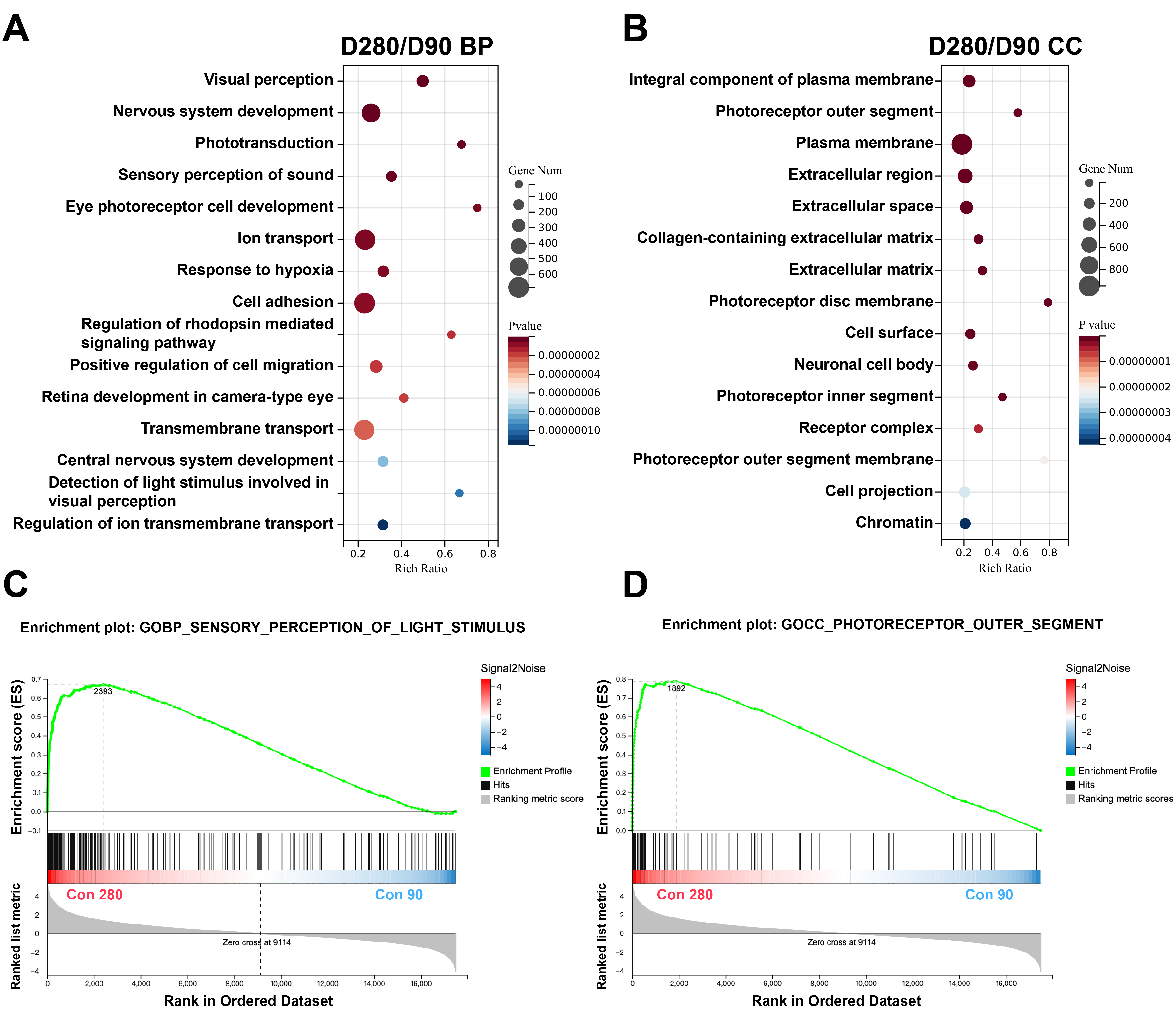
**

**Supplement Fig. 3** Transcriptome analysis for the development of hEROs. (A) Enriched GO terms in BP category in 280-day hEROs, compared to 90-day hEROs. The statistically significant enriched GO terms were ranked by gene numbers. (B) Enriched GO terms in CC category in 280-day hEROs, compared to 90-day hEROs. The statistically significant enriched GO terms were ranked by gene numbers. (C) GSEA analysis showed the enrichment of gene sets associated with sensory perception of light stimulus in 280-day hEROs, compared to 90-day hEROs. (D) GSEA analysis shows the enrichment of gene sets associated with photoreceptor outer segment in 280-day hEROs, compared to 90-day hEROs.





**Supplement Fig. 4** Changes of mRNA levels of the genes related to lipid metabolism and ECM-receptor interaction in 90-day and 280-day hEROs after short-term S protein exposure, compared to control groups. (A) The relative mRNA levels of lipid metabolism-related genes DHCR24, LDLR, INSIG1, and E2F1 in 90-day hEROs after S protein exposure, compared to control groups. (B) The mRNA expression levels of ECM-receptor interaction-related genes FN1, ITGA1, and THBS2 in 280-day hEROs after S protein exposure, compared to control groups. Data are presented as means ± SD. ****P*<0.001, **** *P*<0.0001.
